# Infliximab for the treatment of intravenous immunoglobulin resistant Kawasaki disease complicated by coronary artery aneurysms: a case report

**DOI:** 10.1186/1546-0096-7-3

**Published:** 2009-01-21

**Authors:** Robert J Brogan, Despina Eleftheriou, James Gnanapragasam, Nigel J Klein, Paul A Brogan

**Affiliations:** 1 Department of Paediatric Rheumatology, UCL Institute of Child Health and Great Ormond Street Hospital, 30 Guilford Street, London, UK; 2 Congenital Cardiac Centre, North Wing, Southampton University Hospitals NHS Trust, Tremona Road, Southampton, UK; 3 Department of Infectious disease and Microbiology, UCL Institute of Child Health and Great Ormond Street Hospital, 30 Guilford Street, London, UK

## Abstract

This case report describes an 8 year old boy with IVIG resistant Kawasaki disease complicated by severe bilateral coronary artery aneurysms successfully treated with infliximab, a monoclonal antibody against tumour necrosis factor alpha.

## Background

Kawasaki disease (KD) is associated with the development of systemic vasculitis complicated by coronary and peripheral arterial aneurysms, and myocardial infarction in some patients [1]. KD has an incidence of 8/100 000 children in the UK, and has superseded rheumatic fever in that KD is now the commonest cause of acquired heart disease in children in the United Kingdom and the USA [2]. Treatment with intravenous immunoglobulin (IVIG) and aspirin reduces the incidence of coronary artery lesions (CAL) from approximately 20–40% to <5% [1,2], although recently in the UK we and others have reported an alarmingly higher incidence of CAL despite IVIG therapy, probably relating to delayed diagnosis of KD in the UK [3,4]. It is generally accepted that an as yet undefined infectious trigger in a genetically predisposed individual results in the disease [5].

Despite intensive research into the illness the cause remains unknown, and although there have been significant improvements in diagnosis and treatment of children with the disease there are still a number of important unanswered questions regarding aetiopathogenesis [6], treatment of IVIG resistant cases, and long-term outlook [2]. This case report describes an 8 year old male with severe coronary artery aneurysms (CAA) and ongoing inflammation despite therapy with IVIG, aspirin and corticosteroids, treated successfully with infliximab.

## Case presentation

The patient was an eight year old boy presenting to his local hospital with a three day history of pyrexia, irritability, erythematous rash in the groin, bilateral knee arthritis and generalised arthralgia, and a one day history of bilateral non-purulent conjunctivitis. There was no prior past medical or family history of note and he was fully immunised. Examination revealed red, cracked lips but there was no lymphadenopathy or clinically overt focus of infection. Laboratory investigations at that stage revealed haemoglobin (Hb) 12.6 g/dL (normal reference range 11.5–15.5 g/dL), white cell count (WCC) 10.6 × 10^9^/L (4.5–13.5 × 10^9^/L), platelets 444 × 10^9^/L (150–450 × 10^9^/L), erythrocyte sedimentation rate (ESR) 80 mm/hr (0–10 mm/hr), C-reactive protein (CRP) 143 mg/L (<20 mg/L) and Albumin (Alb) 32 g/L. He was initially treated symptomatically with paracetamol, ibuprofen and broad spectrum antimicrobials, but with no response. All blood cultures and serology for infections were subsequently negative. By day 7 of illness he had persistent spiking fever up to 40.2°C, and developed desquamation of his palms and soles, and severe colicky central abdominal pain without peritonitis. He had also developed worsening arthritis in his ankles, wrists and elbows. Repeat laboratory tests revealed ongoing severe systemic acute phase responses with ESR 99 mm/hr, CRP 185 mg/L, platelets to 512 × 10^9^/L and total WCC 11.5 × 10^9^/L. Ultrasound of abdomen and hips and bone scan revealed no abnormalities.

Rheumatoid factor, anti-nuclear antibody, autoantibodies to extractable nuclear antigens, and anti-neutrophil cytoplasmic antibodies all proved negative. Renal and liver function tests were normal and a bone marrow aspirate demonstrated reactive inflammatory changes with no evidence of malignancy. Electrocardiogram (ECG) revealed an episode of Wenckebach heart block with baseline tachycardia (heart rate 100 beats per minute). Thereafter, echocardiography revealed both right and left coronary artery dilation of 5 mm and a diagnosis of complete Kawasaki disease was confirmed.

On day 14 of illness, the patient was given Intravenous Immunoglobulin (IVIG) 2 g/kg over 12 hours and high dose aspirin. Within 48 hours there was persisting fever and ongoing severe acute phase response (CRP at 206 mg/L, WCC 15.4 × 10^9^/L and platelets 822 × 10^9^/L).

A second dose of IVIG 2 g/kg was administered 17 days after disease onset. Despite this the patient remained febrile with persisting arthritis and irritability. A repeat echocardiogram on day 18 of illness revealed left coronary artery (LCA) dilation of 5.3 mm and right coronary artery (RCA) of 4.3 mm. His Hb dropped to 6.4 g/dL requiring transfusion. The patient had also developed a systolic murmur and repeat echocardiography showed further RCA dilation of 7 mm and LCA dilation of 5 mm.

**Figure 1:**
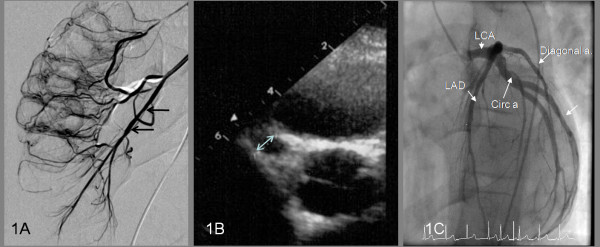
**Arteriographic and echocardiography findings**. (A) Selective visceral arteriogram demonstrating calibre variation and fusiform dilatation of the inferior mesenteric artery (arrows). (B) Echocardiogram on day 34 demonstrated multiple large coronary artery aneurysms including a 7 mm aneurysm affecting the proximal right coronary artery (arrowed). (C) Formal coronary digital subtraction arteriography 9 months after disease onset demonstrating minor ectasia of the left main coronary artery (LCA) and proximal circumflex (Circ. a, arrowed) and proximal left anterior descending artery (LAD, arrowed). This is most noticeable on the LAD just at the origin of the diagonal artery (Diagonal a, arrowed). The right coronary artery was of a normal caliber (not shown).

On day 32 of illness he was transferred to Great Ormond St Hospital for Children for further management. Repeat echocardiography confirmed the severe CAA previously documented and selective visceral arteriography revealed pathological calibre variation of the inferior mesenteric artery (Figure 1A) compatible with medium sized mesenteric artery vasculitis. At this point, he was given intravenous methylprednisolone 30 mg/kg daily for three consecutive days followed by oral prednisolone 1 mg/kg (40 mg) per day for one week thereafter weaning by 5 mg per week. Despite this he had persistent fever, arthritis, abdominal pain and irritability although the CRP had fallen to 25 mg/L. A repeat echocardiogram now showed deterioration in the CAL with overall RCA dilation of 5.4 mm and with a proximal RCA aneurysm of 7 mm (Figure 1B); left main coronary artery dilation of 6.7 mm and left anterior descending artery dilation of 5.9 mm with a distal aneurysm at 6 mm. In view of persisting systemic inflammation and deteriorating CAL on illness day 34 he was given a single dose of infliximab 6 mg/kg after which he became afebrile with his arthritis and arthralgia subsiding within 24 hours. Additionally he was commenced on low molecular weight (LMW) heparin and warfarin aiming to achieve a target International Normalised Ratio (INR) of 2–3 after which the LMW heparin was discontinued, although aspirin was continued at 75 mg once daily.

Thereafter the CAL progressively improved and follow-up echocardiography 5 months after onset of KD revealed partial resolution of his coronary dilation: RCA 3.4 mm and LCA 4 mm proximally and 5.7 mm distally. Formal coronary digital subtraction arteriography 9 months after disease onset demonstrated minor ectasia of the left main coronary artery (LCA) and proximal circumflex and proximal left anterior descending artery. This was most noticeable on the LAD just at the origin of the diagonal artery. The right coronary artery was of a normal caliber (Figure 1C).

## Discussion

Treatment of KD is aimed at reducing inflammation, preventing the formation of CAA and arterial thrombosis [7]. Up to 15% of patients do not respond to a single dose of IVIG and a second dose may be required [2,8], although such IVIG resistant cases may be at higher risk of CAL [2]. Recently it has been suggested that corticosteroids may be used with comparable efficacy to a second dose of IVIG for children who fail to respond to the first dose [9].

Our patient was given his first dose of IVIG on illness day 14, but failed to defervesce after two doses of IVIG. In IVIG resistant KD a number of therapies are reported to be of benefit such as corticosteroid therapy [10], plasma exchange, ulinastatin, and therapy directed at blockade of tumour necrosis factor alpha (TNF-α) [2]. Of these, most attention has focused on corticosteroids either for IVIG resistance [9] or as adjunctive first line treatment in combination with IVIG [11,12]. However, evidence based guidance for alternative treatment if steroids fail (as in our patient) are lacking. Our patient was treated with a single dose of infliximab 6 mg/kg. After the infusion his fever and other systemic symptoms promptly resolved. Encouragingly his severe CAAs began to resolve although we cannot attribute this to infliximab alone. Nonetheless our case highlights the potential for anti-TNF-α therapy in the acute management of CAA in the face of ongoing recalcitrant systemic inflammation.

There are emerging animal data and case reports suggesting efficacy of anti-TNF-α for the treatment of KD [13-21]. The reported clinical cases are summarised in Table 1. Of note is that the use of infliximab for KD is not widely reported in the UK or Europe, most cases described from North America. As yet there have been no published phase three randomized controlled clinical trials of TNF-α blockade in KD. In one retrospective study of seventeen children with IVIG resistant KD infliximab was used successfully with abrupt defervescence in 13/16 febrile patients, with no infusion reactions [18]. Burns et al have also very recently published a phase 2 clinical trial (Table 1) including 16 subjects receiving infliximab that demonstrated that this treatment was safe and well tolerated in patients resistant to IVIG (Table 1) [21]. With IVIG use now coming under increasing scrutiny in the UK [22] use of alternative therapies such as TNF-α blockade are likely to become more widespread, particularly in those with severe CAL with IVIG resistant inflammation.

**Table 1:** Previously published reports of Infliximab for the treatment of IVIG resistant Kawasaki disease.

**Author** **(Reference)**	**Number of cases**	Age* (M/F)	**CAA**	**Treatment pre-infliximab**	**Infliximab dose**	**Outcome**
Weiss et al [14]	1	3 years (M)	Giant CAA	IVIG × 8 MP × 8 Pred PO	5 mg/kg × 3	Resolution of fever post Infliximab; no further progression of CAA
Oishi et al [15]	1	1 month (F)	CAA of RCA and LCA	IVIG × 3 (first two doses at 1 g/kg, 3^rd ^dose at 2 g/Kg) MP × 2 Pred PO Ulinastatin	5 mg/kg × 1	Defervesced within 24 hours of infliximab; developed transient urticaria post infliximab
Girish et al [16]	1	4 years (M)	CAA of RCA and LCA	IVIG × 2 MP × 3	5 mg/kg × 1	Immediate defervescence post infliximab
Burns et al [18]	17	2.6 years (0.12–13.1); (11 M/6F)	12/17	All received at least 2 doses of IVIG; 8/17 MP (1–3 doses)	5 mg/kg × 1 (15/17 patients); 10 mg/kg × 1 (2/17 patients)	13/16 febrile patients defervesced post infliximab; 1 patient died of cardiac arrest related to CAA 53 days after infliximab
Stenbog et al [19]	2	11 weeks (M) 33 weeks (M)	2/2	Both received at least 2 doses of IVIG; MP Pred PO	6 mg/kg × 3	Prompt defervescence and regression of CAA in both cases
O'Connor et al [20]	1	7 weeks (M)	Giant CAA and peripheral gangrene	IVIG × 2 MP × 3	5 mg/kg × 2	Fever resolved after second dose of infliximab; patient received pred PO (for 2 weeks) after infliximab
Burns et al [21]	16	1.7 years (0.7–3.1) (8M/4F) sex not documented in 4 patients receiving infliximab after 2 doses of IVIG	5/16	Single dose of IVIG (n = 12); IVIG × 2 (n = 4)	5 mg/kg × 1	11/12 patients receiving infliximab after a single IVIG dose defervesced within 24 hours; 2/4 patients receiving infliximab after 2 doses of IVIG defervesced; no serious adverse events

M = Male, F = Female; CAA = coronary artery aneurysms; IVIG = intravenous immunoglobulin (2 g/kg unless otherwise stated); MP = methylprednisolone (intravenous pulsed therapy); Pred PO = oral prednisolone; Rca = Right coronary artery; Lca = Left Coronary artery. *Median age (range) for case series.

Since the recognition that IVIG could reduce the morbidity and mortality of KD, treatment of this condition has been protocol driven. Although authorities differ in their advocacy for a variety of treatment protocols, it is likely that the success of therapeutic intervention in KD is due to modulation of the causes and/or propagators of inflammation. As such it is perhaps important to re-evaluate the current prescriptive approach to the management of KD. Is it correct to persist with interventions in a patient in whom there is little evidence of clinical and/or laboratory remission? If ongoing inflammation were damaging to the vasculature, it would seem logical to assess the success (or lack of) of any interventions on the basis of cessation of clinical/laboratory inflammation.

If rapid and effective interruption of inflammation is a primary target of KD therapy, TNF-α blockade may be useful as first line therapy, either in place of or adjunctive to IVIG for the initial treatment of KD. In that context in the UK the cost of a single dose of 2 g/kg IVIG exceeds that of a single dose of infliximab, and therefore infliximab could provide a cheaper alternative to IVIG in the future. As yet, however, we are unaware of any case reports describing the use of infliximab or other anti-TNF-α therapy as first line therapy for KD, and this currently remains an experimental albeit promising novel therapy for IVIG resistant KD.

## Consent

Written informed consent was obtained from the patient for publication of this case report and any accompanying images. A copy of the written consent is available for review by the Editor-in-Chief of this journal.

## Competing interests

The authors declare that they have no competing interests.

## Authors' contributions

RB drafted the manuscript and participated in its design. DE and NK participated in drafting of the manuscript and participated in its design. JG participated in the drafting of the manuscript, carried out the investigations and supplied the images used for the manuscript. PB conceived of the case report, participated in the drafting the manuscript and gave final approval for the version to be submitted for publication.
